# Personality functioning in adolescents with depression: links with childhood maltreatment, psychopathology, self-harm and suicidal ideation

**DOI:** 10.3389/fpsyt.2026.1778390

**Published:** 2026-06-05

**Authors:** Asta Adler, Rasa Barkauskiene, Gabrielė Skabeikytė-Norkienė

**Affiliations:** Institute of Psychology, Vilnius University, Vilnius, Lithuania

**Keywords:** adolescence, depression, identity, personality functioning, self-harm

## Abstract

**Background:**

Adolescence represents a critical developmental period marked by significant vulnerability to depression, a condition with heterogeneous presentations that complicate clinical management. The co-occurrence of personality dysfunction with depression is known to indicate greater severity and poorer prognosis, yet specific features of this comorbidity in adolescent clinical samples require further delineation. This study aimed to compare clinical and psychosocial correlates in depressed adolescents with and without impaired personality functioning.

**Methods:**

The clinical sample comprised 73 adolescents (aged 12–17; 83.6% female) diagnosed with depression or reporting clinically significant depressive symptoms. Participants completed self-report measures assessing personality functioning (LoPF-Q 12–18), childhood maltreatment (CEQ), psychopathology (YSR 11-18), mentalizing capacity (RFQY-8), borderline traits (BPFS-C), and self-harm behavior. Based on LoPF-Q 12-18 T-scores, participants were categorized into two subgroups: depression without personality dysfunction (T ≤ 64; n=20) and depression with personality dysfunction (T ≥ 65; n=53).

**Results:**

Results indicated that adolescents with co-occurring depression and personality dysfunction exhibited significantly lower functioning across all personality domains compared to those with depression alone. This subgroup also reported an earlier onset and higher frequency of self-harm, more severe suicidal ideation, elevated borderline traits, and greater impairments in mentalizing. Furthermore, they demonstrated higher levels of internalizing, affective, conduct, and PTSD symptoms, alongside greater exposure to emotional neglect.

**Conclusion:**

Impaired personality functioning in depressed adolescents is associated with elevated and multifaceted patterns of symptoms, including subjective distress and risky behaviors. These findings underscore the necessity of routinely assessing personality functioning in adolescents with clinically significant depressive symptoms to enable early identification and the development of tailored, integrated interventions for this high-risk subgroup.

## 1 Introduction

Adolescence represents a critical developmental period marked by profound biopsychosocial changes and increased vulnerability to the emergence of mental health disorders (1). Depression commonly emerges during this stage, with approximately 34% of adolescents reporting elevated depressive symptoms and around 8% meeting criteria for Major Depressive Disorder (2). Clinical presentation of depression is notably heterogeneous, varying widely in symptom profiles, developmental pathways, and treatment response (3, 4). This heterogeneity limits the explanatory and clinical utility of symptom-based diagnostic models, particularly during adolescence, when psychopathology is still unfolding.

One major contributor to this heterogeneity is the frequent co-occurrence of depression and personality pathology. Adult studies indicate substantial overlap, with nearly half of individuals with major depressive disorder meeting criteria for a personality disorder and vice versa (5). In such cases, individuals are often understood and treated primarily for depressive symptoms, without sufficient consideration of underlying personality structure and dynamics (6). This tendency may be particularly problematic in adolescence, when personality functioning is still consolidating, and impairments are less likely to be routinely assessed. Recently, this pattern of comorbidity has been conceptualized as complex depression and is associated with greater symptom severity, chronicity, treatment resistance, and elevated risk for self-harm and suicide (7).

Emerging evidence indicates that personality dysfunction is both detectable and clinically meaningful during adolescence, demonstrating levels of continuity and prognostic relevance (8, 9).The Alternative Model for Personality Disorders (AMPD) in DSM-5 provides a developmentally sensitive framework for conceptualizing this pathology through the lens of impaired personality functioning, named as Criterion A. This model assesses deficits in self-functioning (identity, self-direction) and interpersonal functioning (empathy, intimacy) on a continuum (9). Unlike categorical personality disorder diagnoses, Criterion A’s dimensional approach does not assume trait rigidity, making it particularly suitable for identifying emerging dysfunction in adolescence (10). Given the clinical significance of borderline features in adolescent depression, including their association with affective instability, impulsivity, and treatment resistance, we additionally assessed borderline personality features, which within the ICD-11 framework are increasingly understood as a severity specifier of personality dysfunction (11).

Childhood maltreatment is a central transdiagnostic risk factor for both depression and personality pathology. In particular, emotional neglect may subtly but profoundly undermine the development of a coherent sense of self, reflective functioning, and emotion regulation capacities (12). These disruptions are theorized to compromise core domains of personality functioning, such as identity integration and self-direction, thereby increasing vulnerability to more complex and persistent forms of depression (13). Furthermore, recent mediation analyses in adolescent samples demonstrated that childhood maltreatment predicts impaired personality functioning through its effect on both internalizing (e.g., depression, anxiety) and externalizing difficulties (14).

Despite these theoretical links and established adult findings, empirical studies specifically examining personality functioning within adolescent clinical depression samples remain scarce (6, 9). Consequently, it is unclear whether impairments in personality functioning delineate a distinct, high-risk subgroup of depressed adolescents characterized by greater developmental adversity, broader psychopathology, and elevated risk behaviors.

The present study addresses this gap by preliminary examining a clinical sample of adolescents with depression or clinically significant depressive symptoms, comparing those with and without significant personality dysfunction. We hypothesized that adolescents with depression and co-occurring personality dysfunction (the DPD group), compared to adolescents with depression alone (the DO group), would demonstrate: (1) more severe impairments across all domains of personality functioning; (2) greater exposure to childhood maltreatment; (3) higher levels of general psychopathology, borderline features, and mentalizing difficulties; and (4) an increased prevalence and severity of self-harm and suicidal ideation.

## 2 Methods

### 2.1 Participants and procedures

The study sample consisted of 73 adolescents aged 12 to 17 years (M = 15.05, SD = 1.33), of whom 83.6% were female. All participants were recruited from clinical settings – including psychiatric hospitals and private psychological practices – where they were receiving psychological assessment or treatment. At the time of the study, 65.8% of adolescents had been diagnosed with depression by their psychiatrists, while 34.2% of the sample exhibited clinically significant depressive symptoms, as reported by their psychologists during the initial assessment, without a formal diagnosis. The entire sample was characterized by the presence of depressive symptomatology and represents a clinically referred population. In terms of residential background, 47.9% lived in a city, 38.4% in a town, and 9.6% in a rural area. Regarding family structure, 52.1% lived in two-parent households, 23.3% had divorced parents, 5.9% had parents living apart, 8.8% reported that their parents had never been married, and 4.4% lived with a single parent due to the death of the other parent.

Information about the study, participant invitations, and parental consent forms were distributed via mental health clinicians. The study was conducted individually with adolescents currently receiving outpatient and/or inpatient mental health care. Informed consent was obtained from both participants and their legal guardians. Exclusion criteria included the presence of a developmental disorder, such as intellectual disability or a diagnosed autism spectrum condition. The questionnaires were administered by trained researchers, and afterwards all participants received written information about available emotional support resources. Participation was voluntary and no financial compensation was provided.

### 2.2 Measures

Childhood maltreatment experiences were assessed using the Childhood Experiences Questionnaire (CEQ), adapted from the Adverse Childhood Experiences Questionnaire (15). The Lithuanian version consists of 10 items, with two items assessing each of the five types of maltreatment: emotional abuse, emotional neglect, physical abuse, physical neglect, and sexual violence (16). Items related to emotional and physical abuse/neglect are rated on a 5-point Likert scale ranging from 0 (*never*) to 4 (*very often*). Emotional abuse involves harmful hostile acts toward a child’s psyche (e.g., belittling), while emotional neglect involves omissions of needed emotional support (e.g., ignoring), and similarly, physical abuse involves harmful physical acts (e.g., hitting), whereas physical neglect involves omissions of basic care (e.g., failing to provide food). Items on sexual abuse are answered dichotomously (*yes =* 1, *no =* 0). Subscale scores are calculated by summing the respective item responses, and a total score is obtained by summing across all scales, yielding a possible range from 0 to 34. Higher total scores indicate greater exposure to childhood adversity. In the present study, internal consistency for the total scale, as measured by Cronbach’s α was very good (α = .84).

Personality functioning was assessed using the Lithuanian version of the Levels of Personality Functioning Questionnaire, 12–18; (17), which was culturally adapted and validated in Lithuania (18). The questionnaire consists of 97 items rated on a 5-point Likert scale ranging from 0 (*no*) to 4 (*yes*), reflecting a continuum from healthy to impaired personality functioning. Higher scores indicate greater impairment and allow for differentiation between normative and pathological personality development during adolescence. The LoPF-Q 12- 18 yields a total score and four domain scores corresponding to Criterion A of the DSM-5 Alternative Model for Personality Disorders: identity, self-direction, empathy, and intimacy. In line with recent research supporting a unidimensional structure with superior psychometric properties (18, 19), the total score was used in the present study. Internal consistency for the total score was excellent (Cronbach’s α = .90).

Borderline personality features were assessed using the Borderline Personality Features Scale for Children–11 BPFSC-11; (20). The BPFSC-11 is designed to evaluate core features of borderline personality disorder in children aged 9 and older, including adolescents. The scale consists of 11 items reflecting key characteristics of borderline pathology, such as emotional instability, identity disturbance, and interpersonal difficulties. Example items include: *“My feelings about myself change a lot”* and *“I worry that people who are important to me will leave and not come back.”* The scale does not include items related to deliberate self-harm. Responses are rated on a 5-point Likert scale ranging from *“not at all true”* to *“always true.”* A total score is calculated by summing the items, with higher scores indicating greater severity of borderline features. The BPFSC-11 has demonstrated good psychometric properties in clinical adolescent samples (Cronbach’s α = .85; 20). In the current study, internal consistency was also good (Cronbach’s α = .79).

Reflective functioning was assessed using the Reflective Function Questionnaire for Youth RFQY-8; (21). The scale consists of 8 items rated on a 7-point Likert scale, measuring two dimensions: certainty (RFQ - C) and uncertainty (RFQ - U) about one’s own and others’ mental states. Lower RFQ - C scores reflect hypermentalization, while higher RFQ - U scores indicate hypomentalization. The RFQ - C scale measures how confident individuals are in interpreting their own and others’ mental states. For example, strong disagreement with the item “People’s thoughts are a mystery to me” may reflect a tendency toward hypermentalization – an overconfidence in mental state attribution without sufficient evidence (22). In contrast, the RFQ_U scale captures uncertainty and confusion about mental states. For instance, strong agreement with the item *“Sometimes I do things without really knowing why”* reflects hypomentalization, a diminished ability to understand complex internal experiences of oneself and others (22). In this study, internal consistency was acceptable (RFQ -C α = .69; RFQ -U α = .70). The scale has shown good psychometric properties in previous studies across clinical and non-clinical samples (22).

The Youth Self-Report YSR 11–18; (23) was used to assess psychopathological symptoms in adolescents. The study employed an adapted and standardized Lithuanian version of the scale (24). The questionnaire includes 112 items assessing a broad range of emotional and behavioral difficulties, rated on a 3-point Likert scale (0 = not true, 1 = somewhat or sometimes true, 2 = very true or often true), reflecting experiences over the past six months.

Both empirically derived and DSM-oriented scales were utilized in this study. Externalizing and Internalizing problems dimensions demonstrated high internal consistency (respectively, Cronbach’s α = .88 and.88). Additionally, DSM-oriented subscales were included to capture specific symptom dimensions: Affective Problems (α = 0.78), Anxiety Problems (α = 0.71), Somatic Complaints (α = 0.69), Oppositional Defiant Problems (α = 0.69), Conduct Problems (α = 0.76), Post-Traumatic Stress Problems (α = 0.76). These scales provided a dimensional assessment aligned with DSM criteria, allowing for a more nuanced understanding of co-occurring psychopathology in this clinical sample.

Suicidal ideation was evaluated using a single item: *“I think about killing myself, “* which has been shown to be clinically relevant for identifying suicide risk in adolescents.

Self-harming behavior was assessed using items adapted from the Child and Adolescent Self-Harm in Europe (CASE) study. Participants were asked to respond to this section only if they had ever intentionally engaged in self-harm. The first five items collected general information about the behavior: (1) age at first self-harm incident, (2) total number of self-harm episodes, (3) time of most recent self-harm, (4) whether medical help was sought, and (5) whether the act occurred while the participant was alone. An additional eight items assessed potential reasons for self-harm, such as emotional regulation, punishment, or help-seeking motives. These were answered dichotomously (yes/no).

### 2.3 Data analysis

The study included 73 participants clinically diagnosed with depression or clinically significant depressive symptoms, divided into two groups based on personality dysfunction (LoPF-Q 12-18 T-scores T ≤ 64 without personality disfunction; T ≥ 65 with personality dysfunction): 20 (27.4%) adolescents with depression only and 53 (72.6%) – with both depression and personality dysfunction. This threshold was selected based on the instrument’s manual, which defines T-scores between 60 and 64 as indicating ‘elevated risk’ and T-scores of 65 or above as indicating ‘clinically significant’ personality dysfunction (17, 25). A dichotomous grouping was chosen to facilitate comparison between adolescents with and without clinically significant personality dysfunction. Although a dimensional or multi-level approach would better align with current diagnostic frameworks (ICD-11, AMPD), the small sample size and exploratory nature of this study made a categorical distinction more appropriate for primary analyses.

Group comparisons on continuous variables were analyzed using independent samples *t*-tests. Categorical variables were compared using Pearson’s chi-square (*χ*²) tests.

### 2.4 Sample size and sensitivity

To assess whether this sample size provided sufficient statistical power, a *post-hoc* power analysis was conducted using G*Power (26) or an independent two-tailed t-test comparing means between groups. Assuming a medium effect size (Cohen’s d = 0.5), an alpha level of 0.05, and unequal group sizes (20 vs. 53), the analysis indicated a power of 0.72 (72%). While this is slightly below the conventional 80% threshold, it remains acceptable for exploratory research, particularly given the clinical relevance of the subgroup comparisons. Larger samples would be preferable for detecting smaller effects, but the observed group distribution reflects real-world clinical presentations, where comorbid personality dysfunction is more prevalent than depression alone. Thus, while the smaller subgroup (n=20) may limit precision for subtle differences, the sample remains adequate for detecting moderate to large effects.

## 3 Results

Adolescents in the PD group demonstrated significantly lower functioning across all personality domains compared to those in the DO group. Large effect sizes were observed in both the self-functioning dimension (including self-direction and identity; d = -2.15 to -2.09) and the interpersonal dimension (empathy and intimacy; d = -1.31 to -1.84), all with *p* <.001 (see **Table 1**).

**Table 1:** Personality functioning LoPF-Q 12-18 subscales.

Subscale	M (DO)	SD	M (DPD)	SD	T	*p*	Cohen’s d
**Self dimension**	83, 65	22, 36	132, 47	20, 40	-8, 88	<.001	-2, 33
Self-direction	44.78	15.46	72.29	11.64	-8.21	<.001	-2.15
Identity	39.05	9.50	60.59	10.61	-7.95	<.001	-2.09
**Interpersonal dimension**	63, 15	16, 79	99, 09	19, 88	-7, 170	<.001	-1, 88
Empathy	30.65	11.03	47.45	13.43	-5.70	<.001	-1.31
Intimacy	32.75	8.24	52.16	11.26	-8.29	<.001	-1.84

DO, Depression Only (LoPF T-score ≤ 64); DPD, Depression + Personality Dysfunction (LoPF -Q 12-18 T-score ≥ 65). Bolded constructs represent the two higher-order dimensions of personality functioning.

The comparison between the DO and DPD groups revealed large effect sizes across both the Self and Interpersonal dimensions. Specifically, the Self dimension showed a Cohen’s d of –2.33, while the Interpersonal dimension yielded a Cohen’s d of –1.88, indicating that individuals with DPD scored substantially higher on both dimensions compared to those with DO. Although the effect size was numerically larger for the Self dimension in comparison to Interpersonal dimension, a statistical comparison of the two effect sizes revealed that this difference was not statistically significant (z = –1.01, p = .31). Thus, while both dimensions distinguish the groups markedly, the difference in the magnitude of effect between them should be interpreted with caution.

The DPD group reported significantly higher levels of borderline personality features (*p* <.001, d = -1.03). Regarding mentalizing capacity, these individuals also showed significantly greater uncertainty about mental states (*p* = .004, d = -0.80), although no significant difference was found in the certainty scale (*p* = .078) (see **Table 2**).

**Table 2:** Borderline personality features and reflective functioning.

Variable	M (DO)	SD	M (DPD)	SD	T	*p*	Cohen’s d
BPFSC-11 Total	30, 05	7.01	37.49	7.32	-3.92	<.001	-1.03
RFQ_C (Certainty)	1.02	0.76	0.69	0.66	1.79	.078	0.47
RFQ_U (Uncertainty)	0.78	0.70	1.36	0.73	-3.02	.004	-0.80

Among the dimensions of childhood maltreatment, emotional neglect was significantly more prevalent in the DPD group (*p* = .001, d = -0.89). No significant differences between groups were found for emotional or physical abuse, physical neglect, or total maltreatment scores. Small but significant difference between groups was in experience of sexual abuse (*p* = .015, d = -0.38) (see **Table 3**).

**Table 3:** Associations with childhood maltreatment.

Variable	M (DO)	SD	M (DPD)	SD	T	*p*	Cohen’s d
Emotional Neglect	2.05	2.37	3.92	1.99	-3.39	.001	-0.89
Physical Neglect	0.90	2.13	0.60	1.05	0.81	.421	0.21
Emotional Abuse	3.05	2.93	3.00	2.26	0.08	.939	0.02
Physical Abuse	1.40	2.04	1.35	1.73	0.11	.911	0.03
Sexual Abuse	0.20	0.41	0.44	0.70	-1.46	.015	-0.38
Total Score	7.60	8.10	9.31	5.72	-1.01	.318	-0.26

Compared to the DO group, individuals in the DPD group reported significantly higher levels of overall psychopathology, including both internalizing and externalizing symptoms (all *p* <.005, d = -0.70 to -1.28). The DPD group also showed significantly elevated scores in affective problems, anxiety, conduct problems, and PTSD symptoms. No significant differences between groups were found in somatic complaints or oppositional behavior (see **Table 4**).

**Table 4:** Group differences in psychopathology (YSR).

Variable	M (DO)	SD	M (DPD)	SD	T	*p*	Cohen’s d
Total YSR	69.10	28.36	101.17	23.75	-4.86	<.001	-1.28
Internalizing	24.70	12.22	36.52	8.23	-4.74	<.001	-1.25
Externalizing	14.90	8.02	22.44	9.93	-3.03	.003	-0.80
Affective (DSM)	11.10	5.65	18.08	3.69	-6.15	<.001	-1.62
Anxiety (DSM)	3.35	2.89	4.63	3.06	-2.50	.015	-0.66
Somatic Complaints	4.30	2.88	5.01	2.80	-1.61	.113	-0.42
Oppositional	3.70	1.81	4.60	2.57	-1.43	.158	-0.38
Conduct	4.85	3.56	7.71	4.26	-2.66	.010	-0.70
PTSD	13.75	7.01	18.94	3.89	-4.00	<.001	-1.05

A greater proportion of the DPD group endorsed frequent suicidal ideation (57.7% vs. 50%; *p* = .003, χ² = 11.32). They also reported earlier onset of self-harm (*p* = .056), a higher frequency of self-harming behavior (*p* = .015) and were more likely to have engaged in self-harming behavior with the wish to die (*p* = .006) (see **Table 5**).

**Table 5:** Self-Harm and suicidal ideation by personality functioning group.

Outcome	M/proportion (DO)	M/proportion (DPD)	*p*	Effect size
Suicidal thoughts (YSR item 91)	50% report ‘often’	57.7% report ‘often’	.003	V **=** 0.40
Self-harm onset age	13.69	12.48	.056	d = 0.62
Wish to die the reason for self-harm	53.9%	88.1%	.006	d = 0.90
Self-harm frequency	2.92	3.67	.015	d = 0.93

Values represent M means for continuous variables and proportions (%) for categorical variables.

## 4 Discussion

This study provides empirical support for the clinical relevance of assessing personality functioning in adolescents presenting with depression. A striking finding of the present study is that over two-thirds of depressed adolescents recruited from a clinical setting exhibited clinically significant personality dysfunction. This high prevalence is consistent with prior evidence that personality pathology is common among individuals with major depressive disorder (5) and suggests that in clinical populations, personality impairment may be the norm rather than the exception. Viewed from this perspective, depression in clinically referred adolescents may often represent a manifestation of underlying structural vulnerability, an enduring impairment in self- and interpersonal functioning that predisposes individuals to depressive symptoms, poor mentalization, self-harm, and psychopathological complexity.

If personality dysfunction is indeed the rule rather than the exception, the clinically meaningful distinction lies not in its presence or absence, but in its severity and pervasiveness. Accordingly, we compared adolescents with depression and co-occurring clinically significant personality dysfunction (DPD group) to those with depression alone (DO group). Given that groups were formed based on total LoPF-Q 12-18 scores, differences on subscales are partially expected, and future studies should replicate these findings using independently defined diagnostic groups. Nevertheless, the finding that the DPD group demonstrated significantly higher scores across all domains, with deficits in self-functioning, notably identity and self-direction, being especially severe, highlights the pervasiveness of personality impairment in this group. This clinical profile is of considerable importance, as the DPD group constituted the substantial majority (72.6%) of our clinical sample, underscoring the prevalence and clinical significance of personality dysfunction among depressed youth.

The pattern of co-occurring depression and personality dysfunction observed in our DPD group can be understood through two complementary interpretive lenses. First, it aligns with pathoplasticity models, which propose that co-occurring conditions do not operate independently but rather interact to mutually shape one another’s phenotypic expression, clinical course, and overall severity (6). Within this framework, pre-existing or emerging personality dysfunction likely molds how depressive symptoms are experienced, regulated, and interpersonally expressed, while depressive states may, in turn, further erode fragile self-structure and relational capacities (6). The findings are also consistent with longstanding psychodynamic and integrative models that conceptualize depression as arising from, and being embedded within, foundational disturbances in personality structure, specifically in the domains of self-definition (identity, autonomy) and relatedness (empathy, intimacy) (6, 27). This alignment is echoed in recent phenomenological work differentiating three types of adolescent depression, particularly the ‘upset (refusing) mode, ‘ in which personality organization was found to be less integrated (4).

An alternative to the pathoplasticity interpretation is that both the impairments in personality functioning and the complexity of depressive symptomatology observed in our DPD group may reflect a common underlying structural vulnerability. Psychodynamic structural conceptualizations, such as Kernberg’s model of personality organization and the Operationalized Psychodynamic Diagnosis for Children and Adolescents (OPD-CA-2), posit those structural capacities - including identity formation, affect regulation, and interpersonal relatedness -constitute a foundational matrix that shapes symptomatic presentations across diagnostic categories (28–30). From this perspective, the pervasive deficits in self- and interpersonal functioning in the DPD group are not merely co-occurring conditions interacting with depression, but rather reflect a more fundamental disturbance in personality organization that manifests as both personality dysfunction and greater clinical complexity. This structural interpretation offers a parsimonious account for the high prevalence of personality dysfunction in our clinical sample and aligns with the view that depression in clinically referred adolescents may often represent an expression of underlying structural vulnerability rather than a discrete episodic condition.

These two interpretive lenses, pathoplasticity and structural vulnerability as transdiagnostic factor, need not be mutually exclusive. Rather, they may operate at different levels of explanation, with pathoplasticity describing dynamic interactions between conditions and structural vulnerability capturing the deeper organizational substrate from which both arise. Regardless of which lens is emphasized, both perspectives converge on the same clinical conclusion: assessing personality functioning is essential for understanding the complexity and severity of depression in adolescents.

The DPD group exhibited significantly more borderline personality features and demonstrated greater uncertainty in understanding mental states, a pattern indicative of hypomentalization. This finding aligns with extant literature showing that borderline pathology in adolescents is correlated with deficits in mentalization, problematic attachment relationships, and elevated levels of both internalizing and externalizing psychopathology, including risk-taking, self-harm, and depressive symptomatology (8, 31). Notably, the absence of significant group differences in hypermentalization (i.e., RFQ-C certainty scores) suggests that uncertainty-based mentalizing deficits may represent a particularly salient feature of complex adolescent depression. The non-specificity of this finding is consistent with research positioning hypermentalizing as a correlate of general psychopathology rather than a disorder-specific deficit (32). However, this interpretation must be considered alongside psychometric evidence suggesting the RFQ-8, from which the certainty subscale is derived, may function as a unidimensional measure, potentially limiting its ability to distinctly capture hypermentalization as a separate construct (33). Moreover, the group size imbalance (DPD: n=53, DO: n=20) and *post-hoc* power of.72 in our study indicate insufficient power to detect smaller or more nuanced between-group differences, such as hypermentalization.

Our study revealed no significant differences in overall childhood maltreatment exposure between adolescents with depression only (DO) and those with depression and personality dysfunction (DPD), with the notable exception of emotional neglect. This finding is clinically significant, as childhood maltreatment is a central transdiagnostic risk factor for both depression and personality pathology. Specifically, emotional neglect may subtly but profoundly undermine the development of a coherent sense of self, reflective functioning, and emotion regulation capacities (12).

The specificity of emotional neglect as the sole maltreatment domain differentiating the DPD and DO groups resonates with Blatt’s concept (27, 34) of anaclitic depression, in which early relational disruption, particularly emotional deprivation, is conceptualized to impair the development of a coherent sense of self and capacity for relatedness. Within Blatt’s framework, such anaclitic vulnerabilities, characterized by fears of being abandoned and unloved, are distinct from introjective pathways organized around self-criticism and failure, which may be more closely tied to overt abuse or criticism. Our finding that the DPD group exhibited pervasive deficits in self- and interpersonal functioning aligns with this anaclitic configuration, suggesting that emotional neglect may represent a specific developmental pathway to personality dysfunction in adolescent depression. This also aligns with recent developmental models suggesting that maltreatment, particularly neglect, impairs personality structure through the pathway of internalizing and externalizing psychopathology (14), which our DPD group also exhibited in elevated form.

The broader psychopathological burden observed among DPD adolescents, including elevated internalizing, externalizing, and trauma-related symptoms, reflects a generalized vulnerability captured by impairments in personality functioning (35). The absence of significant DPD vs. DO group differences in somatic complaints and oppositional behavior may appear to conflict with the finding of impaired mentalization in the DPD group. However, this pattern may reflect the specific nature of mentalization deficits in this sample. Impairments in mentalization are often associated with externalizing behaviors and emotional dysregulation (11). Yet, the DPD group in our study exhibited a profile characterized more prominently by deficits in self-functioning (identity, self-direction) and hypomentalization rather than oppositional behavioral problems and their personality pathology may, to a greater extent, reflect an internalizing, rather than an externalizing, dimension of personality disorder. It is possible that the personality dysfunction in this group manifests as internalizing difficulties rather than somatic complaints or oppositional behaviors. Alternatively, the relatively modest sample size and group imbalance may have limited power to detect differences in these specific domains, particularly if effect sizes were small.

Most concerning, the DPD subgroup exhibited earlier onset and greater severity of self-harming behaviors, higher suicidal intent, and more frequent suicidal ideation. This is consistent with prior findings that identify complex depression, marked by personality dysfunction, as carrying a greater risk for self-injurious behavior and suicidality (7, 8). The DPD group showed pronounced instability in identity and goal-directedness. This pattern likely drives the chronicity, severity, and treatment resistance that characterize complex depression (7, 12, 36). Concurrently, the significant impairments in interpersonal functioning reflect the dysfunctional relational patterns and heightened interpersonal stress reactivity theorized to perpetuate emotional dysregulation (37).

Clinically, these findings emphasize the importance of routine assessment of personality functioning in depressed adolescents, allowing for more precise, developmentally informed case formulation and risk management. While treatment outcomes were not evaluated in the present study, the identified profile aligns with the theoretical targets of integrative interventions, particularly those focused on identity consolidation, reflective functioning, and interpersonal relatedness. Consequently, assessing and therapeutically targeting personality domains may be necessary to disrupt maladaptive developmental pathways and improve outcomes for adolescents with complex clinical presentations (38).

Importantly, the relevance of assessing personality functioning is not unique to depression. Impairments in self- and interpersonal functioning are associated with a wide range of mental disorders, including other internalizing disorders (e.g., anxiety disorders) as well as externalizing disorders (e.g., conduct disorder, substance use) (9, 11, 39). From a transdiagnostic perspective, personality dysfunction may represent a shared vulnerability factor contributing to the severity, chronicity, and complexity of various clinical presentations. Thus, while our study demonstrates the utility of assessing personality functioning in depressed adolescents, this relevance likely extends to other psychiatric populations.

Several limitations warrant consideration. The cross-sectional design precludes causal inference, and the modest sample size—particularly the imbalance between groups (DPD: n=53, DO: n=20)—limited statistical power to detect smaller or more nuanced differences. The sample was predominantly female, limiting generalizability to males, and reliance on self-report measures may introduce bias. Our dichotomous grouping approach, while clinically meaningful, does not fully capture the dimensional nature of personality functioning as conceptualized in ICD-11 and the AMPD and it would be important to address in future research. Additionally, we did not systematically screen for bipolar spectrum disorders, which frequently co-occur with affective instability and borderline features; given that our DPD group exhibited elevated borderline features, undetected bipolar spectrum conditions may be present. The use of gold-standard diagnostic instruments, such as semi-structured clinical interviews (e.g., K-SADS or SCID), is also needed to confirm participant diagnoses and enhance validity.

Future longitudinal research with larger, more diverse samples should incorporate multi-informant and behavioral assessments, examine graded approaches to personality functioning (e.g., four to five levels of impairment), and include systematic screening for bipolarity to clarify developmental pathways and inform targeted interventions.

In conclusion, the high prevalence of personality dysfunction in this clinical sample suggests that depression in referred adolescents often reflects an underlying structural vulnerability rather than a discrete episodic condition. Nevertheless, clinically significant personality dysfunction delineates a subgroup with even greater impairments in mentalization, psychopathological complexity, and risk for self-harm and suicide. Assessing personality functioning, particularly deficits in identity, self-direction, and relatedness, offers a valuable framework for identifying vulnerable youth and tailoring interventions to address the foundational impairments underlying their depression.

## Data Availability

The raw data supporting the conclusions of this article will be made available by the authors, without undue reservation.
